# Synthesis and crystal structure of ethyl 2-(1,3-benzo­thia­zol-2-yl)-1-oxo-1*H*-pyrido[2,1-*b*][1,3]benzo­thia­zole-4-carboxyl­ate

**DOI:** 10.1107/S2056989026000204

**Published:** 2026-01-20

**Authors:** Heba A. Elboshi, Rasha A. Azzam, Galal H. Elgemeie, Peter G. Jones

**Affiliations:** a Chemistry Department, Faculty of Science, Helwan University, Cairo, Egypt; b Institut für Anorganische und Analytische Chemie, Technische Universität Braunschweig, Hagenring 30, D-38106 Braunschweig, Germany; Vienna University of Technology, Austria

**Keywords:** crystal structure, benzo­thia­zole, hydrogen bonds, π stacking

## Abstract

The mol­ecule of the title compound, C_21_H_14_N_2_O_3_S_2_, is approximately planar except for the terminal carbon atom of the ethyl group. The planarity is promoted by two short intra­molecular S⋯O=C contacts and one intra­molecular ‘weak’ C—H⋯O=C hydrogen bond. The two thia­zole rings show some appreciable differences in bond lengths and angles, associated with their different annelation patterns. The mol­ecular packing involves one ‘weak’ hydrogen bond of the type C—H⋯O=C, which links the mol­ecules in zigzag chains *via* a 2_1_ screw axis along [010]. Additionally, ‘stacked’ pairs of mol­ecules, necessarily with parallel ring systems, are related by an inversion operator, and two π contacts C—H⋯π and C=O⋯π are observed.

## Abstract 2

Two short intra­molecular S⋯O=C contacts and one intra­molecular ‘weak’ C—H⋯O=C hydrogen bond cause most of the mol­ecule to be approximately planar. The main secondary inter­actions are a ‘weak’ hydrogen bond, stacked pairs of mol­ecules and a C—H⋯π contact.

## 1. Chemical context

A central objective in the field of medicinal chemistry is the design and development of novel therapeutic mol­ecules to treat infections (Desai *et al.*, 2013 ▸; Li *et al.*, 2000 ▸). Numerous patents and scientific studies have shown that benzo­thia­zole scaffolds have exceptional biological properties (Gill *et al.*, 2015 ▸), and several medications with such heterocyclic rings have been found to have a wide range of pharmaceutical applications and biological activities (Catalano *et al.*, 2021 ▸). In particular, anti­bacterial, anti­viral, anti­fungal, and anti-inflammatory properties have been established for benzo­thia­zole compounds (Azzam *et al.*, 2020 ▸).

It is important to note that the biological actions of benzo­thia­zole derivatives depend on their exact mol­ecular structure, functional groups, and the biochemical path or enzyme they inter­act with (Kamal *et al.*, 2015 ▸). Furthermore, the pharmacokinetic and pharmacodynamic characteristics of benzo­thia­zole-based medications must be carefully taken into account throughout development (Al-Tel *et al.*, 2011 ▸). To maximize their biological activity and minimize any related toxicity, researchers are still investigating and altering the mol­ecular and crystal structures of benzo­thia­zole derivatives (Keri *et al.*, 2015 ▸).

**Figure 1:**
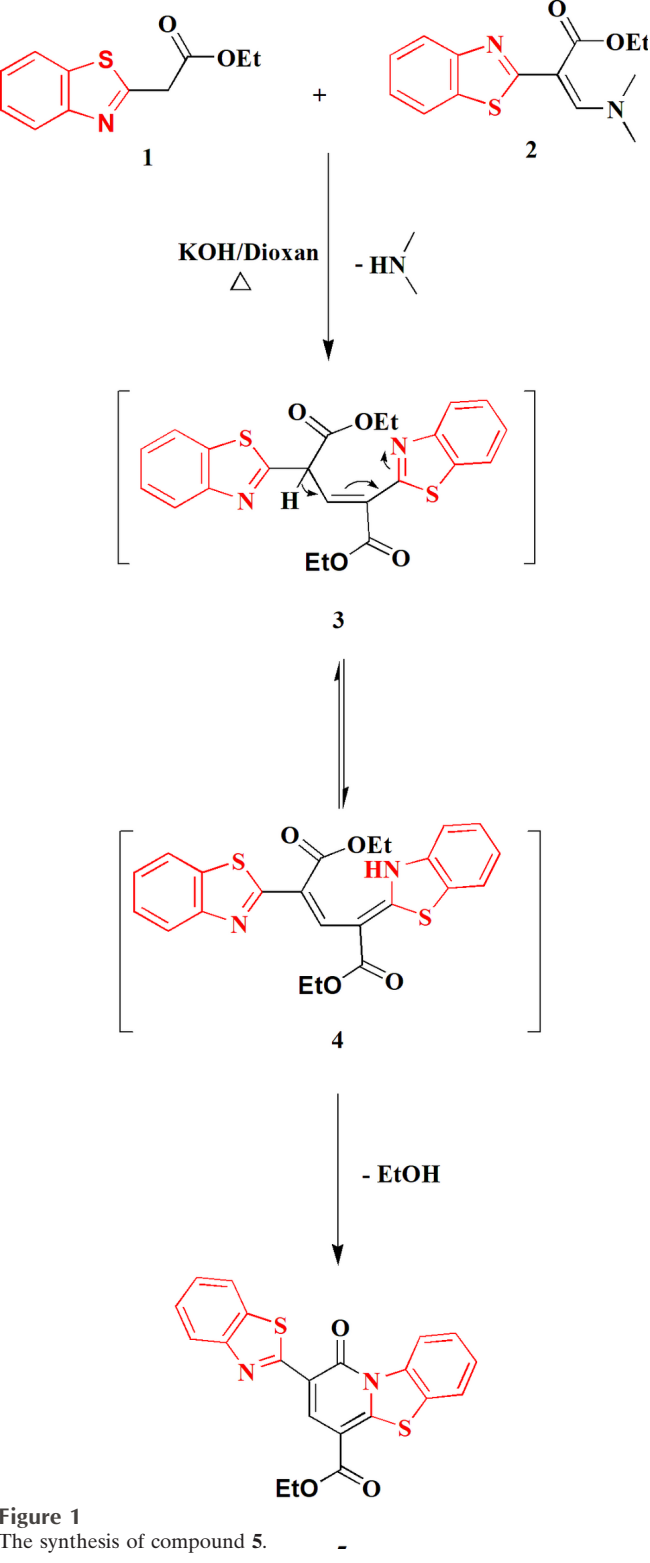
The synthesis of compound **5**.

We have recently synthesized some novel heterocyclic compounds with significant biological activities by incorporating a benzo­thia­zole moiety (Elgemeie *et al.*, 2022 ▸). One such compound, with both beneficial optical properties and biological activity, was a benzo­thia­zole with substituted coumarin residues (Abdallah *et al.*, 2023 ▸). Novel coumarin-benzo­thia­zole compounds, discovered by us, are now being utilized as laser dyes for medical purposes (Elgemeie, 1989 ▸). Additionally, we have synthesized novel benzo­thia­zole-based heterocycles that had noteworthy fluorescence properties and biological significance (Azzam *et al.*, 2022 ▸). In this paper, we provide a novel approach to the synthesis of a condensed benzo­thia­zole-pyridine derivative by reacting ethyl 2-benzo­thia­zolyl acetate **1** with 2-(benzo[*d*]thia­zole-2-yl)-3-(di­methyl­amino)­prop-2-enoate **2** in refluxing KOH/dioxane for two hours; the product, ethyl 2-(1,3-benzo­thia­zol-2-yl)-1-oxo-1*H*-pyrido[2,1-*b*][1,3]benzo­thia­zole-4-carboxyl­ate **5**, was produced in acceptable yield (Fig. 1 ▸). To establish the mol­ecular structure of **5** unambiguously, its crystal structure was determined and is presented here.
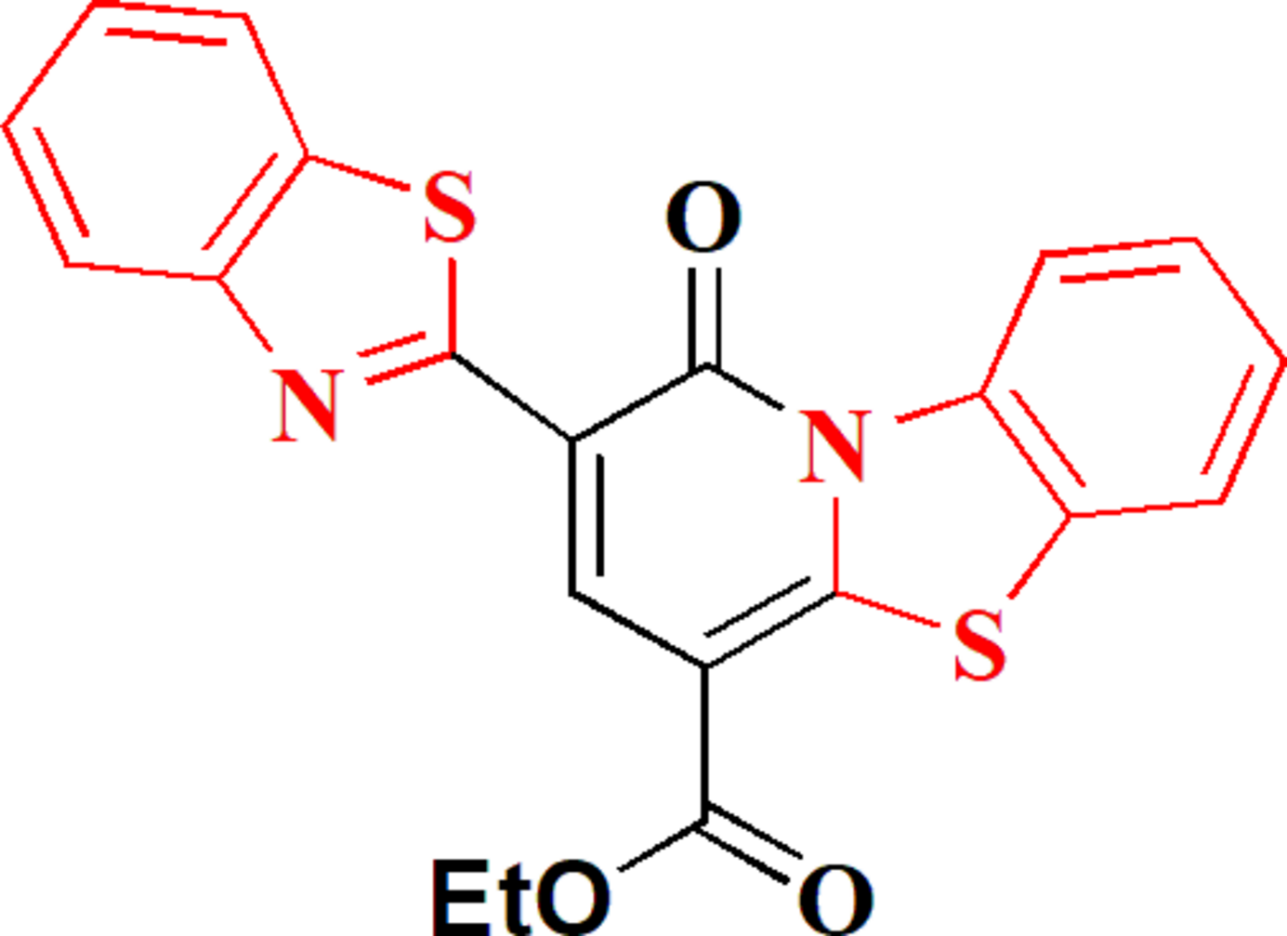


## 2. Structural commentary

**Figure 2:**
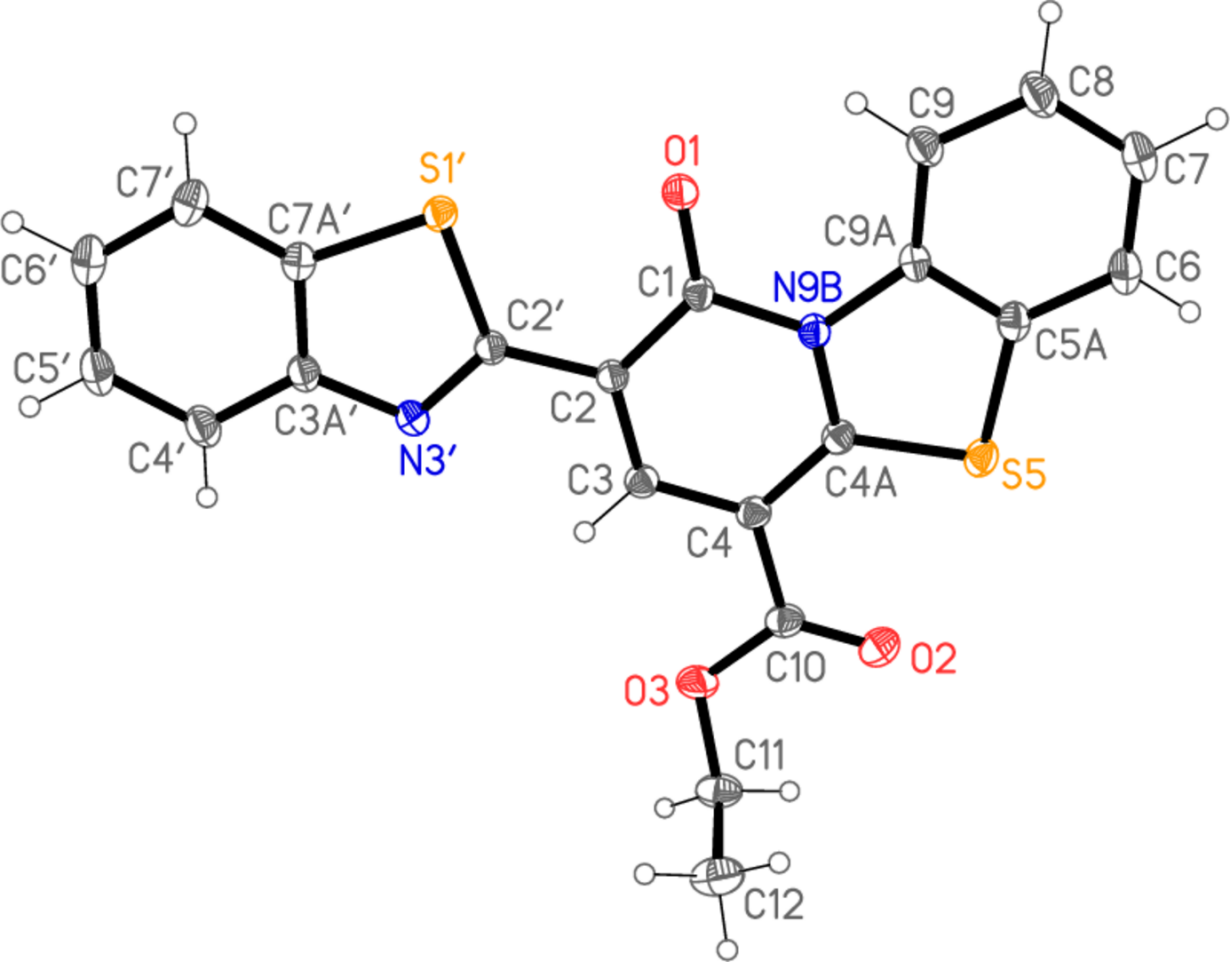
The mol­ecular structure of compound **5** in the crystal; ellipsoids correspond to 50% probability levels.

The mol­ecular structure of compound **5** is shown in Fig. 2 ▸; selected bond lengths and angles are collated in Table 1 ▸. Both the bicyclic and tricyclic ring systems contain a five-membered thia­zole ring, but these show some significant differences in bond lengths and angles, some associated with the additional annelated ring in the tricyclic system. Thus the largest difference in bond lengths is for the bonds C4*A*—N9*B* = 1.3750 (11) Å, *cf.* C2′—N3′ = 1.3106 (11) Å, the former bond being annelated but the latter non-annelated and thus formally closer to a double bond. Similarly, the angle S5—C4*A*—N9*B* = 112.92 (8)° is appreciably narrower than S1′—C2′—N3′ = 115.89 (6)° (but in fact all related pairs of angles differ by some 1.5–3.5°). The bond length C2—C2′ between the ring systems is 1.4619 (11) Å. The exocyclic S—C—C bond angles of the ring systems are all appreciably greater than 120°, the largest being S1′—C7*A*′—C7′ = 128.87 (7)°, but this is normal for such systems. The angle S1′—C2′—C2, not constrained by being within a ring system, is much smaller at 122.73 (6)°.

**Figure 3:**
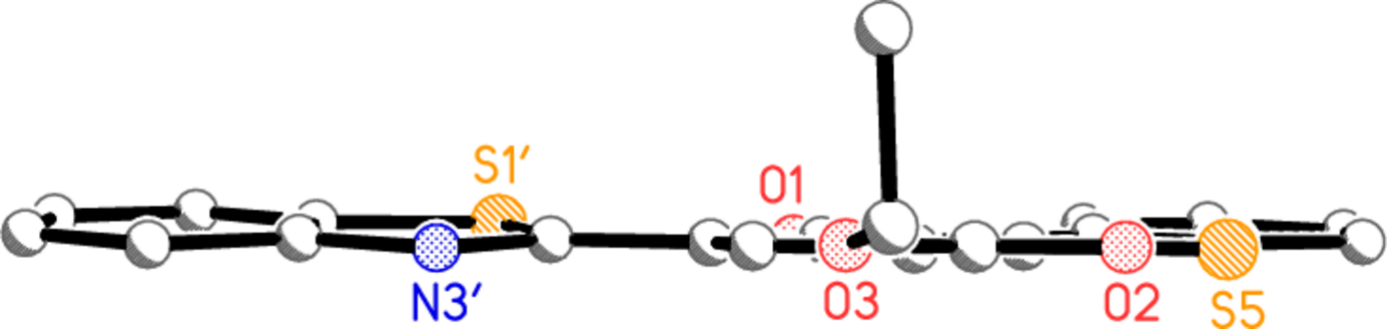
The mol­ecule of **5** viewed ‘edge-on’. Hydrogen atoms are omitted; radii are arbitrary.

Both ring systems are planar to a good approximation (r.m.s. deviations 0.008 Å for the bicyclic and 0.024 Å for the tricyclic system), and, despite the apparent possibility of free rotation about the formally single bond C2—C2′, the inter­planar angle between the ring systems is only 3.12 (3)°, meaning that much of the mol­ecule is planar (Fig. 3 ▸). Associated with this are the short contacts S1′⋯O1 2.7241 (7), S5⋯O2 2.7146 (9) and H9⋯O1 2.24 Å. We have noted such short intra­molecular S⋯O contacts before in related heterocyclic systems, and in one recent paper presented a brief database review of such contacts (Elgemeie *et al.*, 2025 ▸).

## 3. Supra­molecular features

**Figure 4:**
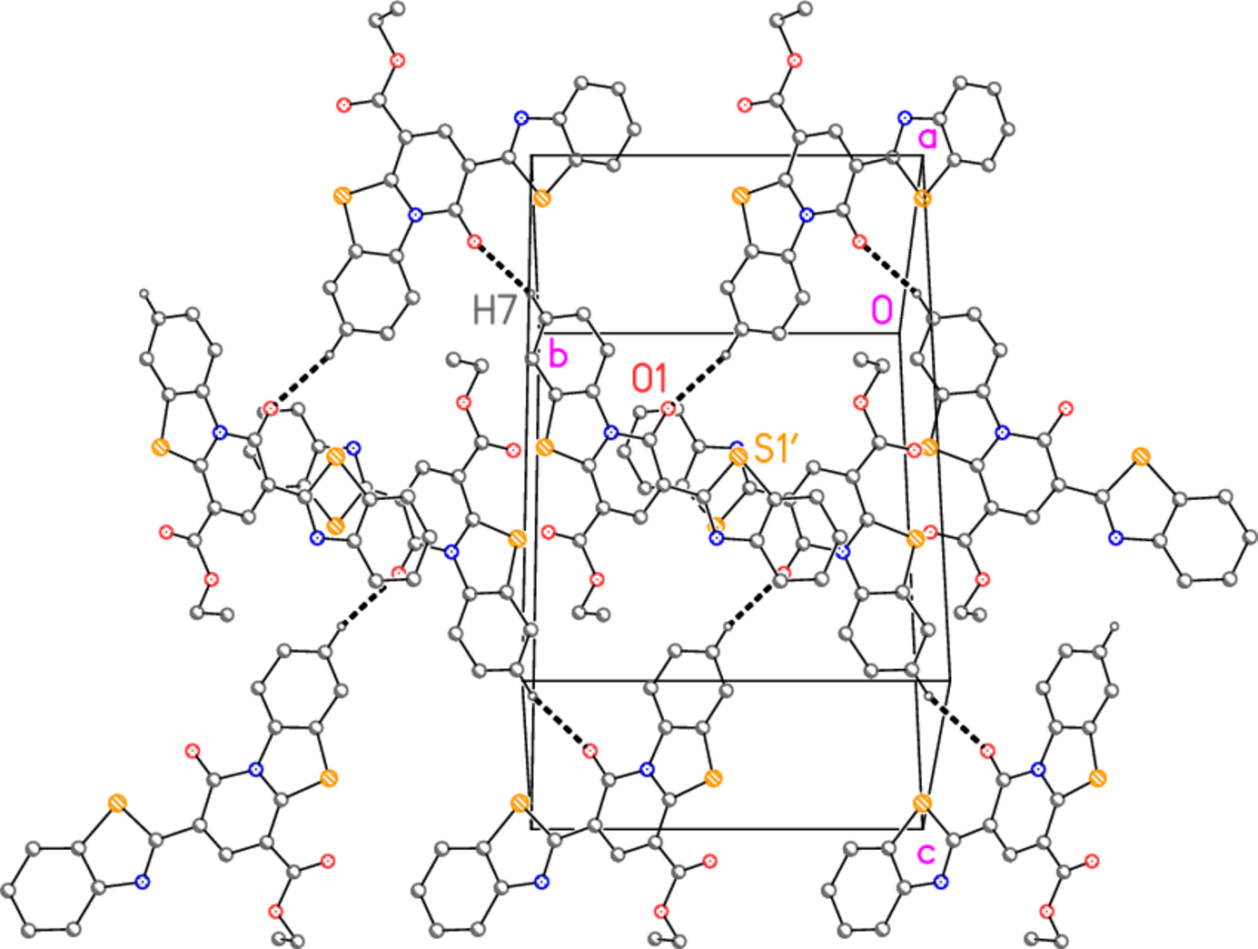
Two zigzag chains of compound **5** parallel to the *b* axis, centred on the region *y* ≃ 0.75. The view direction is approximately perpendicular to the *bc* plane. The mol­ecules are linked *via* a 2_1_ screw axis by a ‘weak’ hydrogen bond (Table 2 ▸). Hydrogen atoms not involved in hydrogen bonding are omitted.

**Figure 5:**
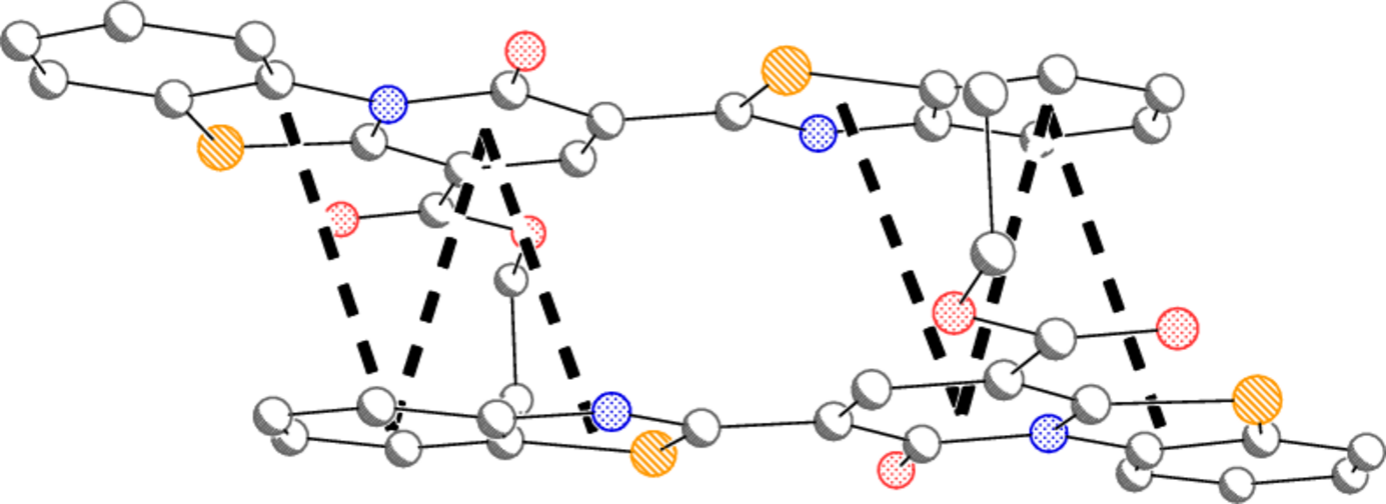
Two neighbouring mol­ecules of compound **5** related by the inversion operator 1 − *x*, 1 − *y*, 1 − *z*. Short contacts between ring centroids are indicated by thick dashed lines (see text). Hydrogen atoms are omitted.

The mol­ecular packing involves just one hydrogen bond, the ‘weak’ contact H7⋯O1 via a 2_1_ screw axis (Fig. 4 ▸, Table 2 ▸). Apart from this, there are several short contacts between ring centres of gravity (*Cg*) of pairs of mol­ecules, necessarily with parallel ring systems, related by the inversion operator 1 − *x*, 1 − *y*, 1 − *z* (Fig. 5 ▸). Denoting the rings from left to right in Fig. 2 ▸ as *A*–*E*, the contacts are: *CgA*⋯*CgC =* 3.5077 (5), *CgA*⋯*CgD* = 3.6392 (5) and *CgB*⋯*CgC* = 3.6371 (5) Å, with slippages of 0.98, 1.37 and 1.33 Å, respectively. The other two short contacts are the probable C—H⋯π inter­action C6—H6⋯*CgA* = 2.49 Å (*via* the glide plane operator −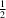 + *x*, 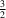 − *y*, −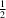 + *z* and with angle 151° at H6) and O2⋯*CgD* = 3.3684 (10) Å (1 − *x*, 2 − *y*, 1 − *z*, with C10—O2⋯*CgD* 89.4°). Attempts to combine more than one set of these contacts in one figure lead to complex three-dimensional diagrams that are difficult to inter­pret.

## 4. Database survey

The search of version 6.00 of the Cambridge Database (Groom *et al.*, 2016 ▸) employed the routine ConQuest (Bruno *et al.*, 2002 ▸; version 2025.1.1). Structures with the same tricyclic ring system as compound **5** were sought (any bond orders, coordination numbers 2 for the sulfur atom and 3 for all other atoms). Seven hits were found, two of which were closely related to **5**, namely 2-(1,3-benzo­thia­zol-2-yl)-4-(furan-2-carbon­yl)-1-oxo-1*H*-pyrido[2,1-*b*][1,3]benzo­thia­zol-3-yl furan-2-carboxyl­ate toluene solvate and 2-(1,3-benzo­thia­zol-2-yl)-4-benzoyl-1-oxo-1*H*-pyrido[2,1-*b*][1,3]benzo­thia­zol-3-yl benzo­ate (refcodes NOTKAM and NOTKEQ; Lystsova *et al.*, 2023 ▸). Both of these (room temperature) structures have oxo functions and 1,3-benzo­thia­zol-2-yl substituents at the same atoms as **5**; they also have closely similar bond lengths between the ring systems [1.463 (3) and 1.464 (5), 1.465 (5) Å] and similar intra­molecular S⋯O contacts that again lead to approximate coplanarity of the ring systems. The structure of the ionic compound 1-amino-2-(1,3-benzo­thia­zol-2-yl)-3*H*-pyrido[2,1-*b*][1,3]benzo­thia­zol-3-iminium chloride methanol solvate (REZVUQ; Chen *et al.*, 2018 ▸) also contains analogously linked bi- and tricyclic systems, but no C=O function corresponding to that of **5**.

## 5. Synthesis and crystallization

Ethyl 2-benzo­thia­zolyl acetate **1** (10 mmol) was added to a stirred solution of 2-(benzo[*d*]thia­zole-2-yl)-3-(di­methyl­amino)­prop-2-enoate **2** (10 mmol) in dry dioxane (30 ml) containing potassium hydroxide (10 mmol) and the reaction mixture was refluxed for 2 h. The precipitate of **5** thus formed was filtered off from the hot solution and recrystallized from ethanol.

Yellow solid (yield 50%), m.p. > 603 K; IR (KBr, cm^−1^): *υ* 3061 (Ar—CH), 1665, 1639 (2C=O); ^1^H NMR (400 MHz, DMSO-*d_6_*): δ = 1.42 (*t*, *J* = 8.0 Hz, 3H, C*H*_3_-CH_2_), 4.14 (q, *J* = 7.2 Hz, 2H, CH_3_-C*H*_2_), 7.45 (t, *J* = 7.2 Hz, 1H, benzo­thia­zole-H), 7.56 (*t*, *J* = 7.2 Hz, 1H, benzo­thia­zole-H), 7.70 (*m*, 2H, benzo­thia­zole-H), 8.08 (*d*, *J* = 10.0 Hz, 1H, benzo­thia­zole-H), 8.15 (*d*, *J* = 10.8 Hz, 1H, benzo­thia­zole-H), 8.26 (*d*, *J* = 7.0 Hz, 1H, benzo­thia­zole-H), 9.28 (*s*, 1H, CH-pyridine), 9.34 (*d*, *J* = 9.2 Hz, 1H, benzo­thia­zole-H); Analysis: calculated for C_21_H_14_N_2_O_3_S_2_ (406.48): C 62.05, H 3.47, N 6.89. Found: C 61.99, H 3.44, N 6.88%.

## 6. Refinement

Details of data collection and structure refinement are summarized in Table 3 ▸. Data were collected at 110 K because the crystals cracked at the standard temperature of 100 K, presumably because of a phase change.

The tricyclic ring system was assigned the standard IUPAC numbering; the benzo­thia­zole system bonded to it was also numbered in the standard fashion, but with added primes (’). The methyl group was refined as an idealized rigid group with C—H = 0.98 Å, H—C—H = 109.5°, allowed to rotate but not tip (AFIX 137). Other hydrogen atoms were included using a riding model starting from calculated positions (C—H_arom_ = 0.95, C—H_methyl­ene_ = 0.98 Å). The *U*(H) values were fixed at 1.5 × *U*_eq_ of the parent carbon atoms for the methyl group and 1.2 × *U*_eq_ for the other hydrogen atoms.

There is one significant peak of residual electron density, namely 1.62 e Å^−3^ near S5. We speculate that it may represent a small amount of contamination by a compound containing a different, but unidentified, heterocyclic system. We note that the peak size is only 0.75 e Å^−3^ if the data are cut to the IUCr limit of 0.84 Å resolution; one cosmetic disadvantage of structures determined to higher resolution is that the sizes of anomalous features in the residual density are magnified, because the Fourier syntheses are summed over a much larger number of reflections.

## Supplementary Material

Crystal structure: contains datablock(s) I, global. DOI: 10.1107/S2056989026000204/wm5782sup1.cif

Structure factors: contains datablock(s) I. DOI: 10.1107/S2056989026000204/wm5782Isup2.hkl

Supporting information file. DOI: 10.1107/S2056989026000204/wm5782Isup3.cml

CCDC reference: 2521548

Additional supporting information:  crystallographic information; 3D view; checkCIF report

## Figures and Tables

**Table 1:** Selected geometric parameters (Å, °)

C1—O1	1.2292 (10)	C9*A*—N9*B*	1.4168 (11)
C2—C2′	1.4619 (11)	S1′—C7*A*′	1.7287 (9)
C4*A*—N9*B*	1.3750 (11)	S1′—C2′	1.7553 (8)
C4*A*—S5	1.7250 (8)	C2′—N3′	1.3106 (11)
S5—C5*A*	1.7403 (10)	N3′—C3*A*′	1.3831 (12)
C5*A*—C9*A*	1.3994 (12)	C3*A*′—C7*A*′	1.4067 (13)
			
N9*B*—C4*A*—S5	112.92 (6)	C7*A*′—S1′—C2′	88.77 (4)
C4—C4*A*—S5	126.17 (6)	N3′—C2′—S1′	115.89 (6)
C4*A*—S5—C5*A*	90.34 (4)	C2—C2′—S1′	122.73 (6)
C6—C5*A*—S5	125.86 (7)	C2′—N3′—C3*A*′	110.29 (7)
C9*A*—C5*A*—S5	112.64 (6)	N3′—C3*A*′—C7*A*′	115.21 (7)
C5*A*—C9*A*—N9*B*	110.92 (7)	C7′—C7*A*′—S1′	128.87 (7)
C4*A*—N9*B*—C9*A*	113.15 (7)	C3*A*′—C7*A*′—S1′	109.83 (6)

**Table 2:** Hydrogen-bond geometry (Å, °)

*D*—H⋯*A*	*D*—H	H⋯*A*	*D*⋯*A*	*D*—H⋯*A*
C9—H9⋯O1	0.95	2.24	2.8116 (11)	118
C7—H7⋯O1^i^	0.95	2.42	3.3470 (12)	164

Symmetry code: (i) 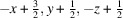.

**Table 3:** Experimental details

Crystal data	
Chemical formula	C_21_H_14_N_2_O_3_S_2_
*M*_r_	406.46
Crystal system, space group	Monoclinic, *P*2_1_/*n*
Temperature (K)	110
*a*, *b*, *c* (Å)	8.7402 (2), 12.0342 (3), 17.2069 (4)
β (°)	100.847 (2)
*V* (Å^3^)	1777.51 (7)
*Z*	4
Radiation type	Mo *K*α
μ (mm^−1^)	0.33
Crystal size (mm)	0.17 × 0.13 × 0.04

Data collection	
Diffractometer	XtaLAB Synergy
Absorption correction	Multi-scan (*CrysAlis PRO*; Rigaku OD, 2024 ▸)
*T*_min_, *T*_max_	0.717, 1.000
No. of measured, independent and observed [*I* > 2σ(*I*)] reflections	138468, 9507, 7763
*R*_int_	0.041
θ values (°)	θ_max_ = 38.3, θ_min_ = 2.4
(sin θ/λ)_max_ (Å^−1^)	0.873

Refinement	
*R*[*F*^2^ > 2σ(*F*^2^)], *wR*(*F*^2^), *S*	0.039, 0.114, 1.04
No. of reflections	9507
No. of parameters	254
H-atom treatment	H-atom parameters constrained
Δρ_max_, Δρ_min_ (e Å^−3^)	1.62, −0.48

Computer programs: *CrysAlis PRO* (Rigaku OD, 2024 ▸), *SHELXT* (Sheldrick, 2015*a* ▸), *SHELXL* (Sheldrick, 2015*b* ▸), *XP* (Bruker, 1998 ▸) and *publCIF* (Westrip, 2010 ▸).

## supplementary crystallographic information

### Ethyl 2-(1,3-benzothiazol-2-yl)-1-oxo-1H-pyrido[2,1-b][1,3]benzothiazole-4-carboxylate Crystal data

**Table:**

C_21_H_14_N_2_O_3_S_2_	*F*(000) = 840
*M**_r_* = 406.46	*D*_x_ = 1.519 Mg m^−^^3^
Monoclinic, *P*2_1_/*n*	Mo *K*α radiation, λ = 0.71073 Å
*a* = 8.7402 (2) Å	Cell parameters from 74718 reflections
*b* = 12.0342 (3) Å	θ = 2.4–38.4°
*c* = 17.2069 (4) Å	µ = 0.33 mm^−^^1^
β = 100.847 (2)°	*T* = 110 K
*V* = 1777.51 (7) Å^3^	Tablet, yellow
*Z* = 4	0.17 × 0.13 × 0.04 mm

### Ethyl 2-(1,3-benzothiazol-2-yl)-1-oxo-1H-pyrido[2,1-b][1,3]benzothiazole-4-carboxylate Data collection

**Table:**

XtaLAB Synergy diffractometer	9507 independent reflections
Radiation source: micro-focus sealed X-ray tube, PhotonJet (Mo) X-ray Source	7763 reflections with *I* > 2σ(*I*)
Mirror monochromator	*R*_int_ = 0.041
Detector resolution: 10.0000 pixels mm^-1^	θ_max_ = 38.3°, θ_min_ = 2.4°
ω scans	*h* = −15→14
Absorption correction: multi-scan (CrysAlisPro; Rigaku OD, 2024)	*k* = −20→20
*T*_min_ = 0.717, *T*_max_ = 1.000	*l* = −29→29
138468 measured reflections	

### Ethyl 2-(1,3-benzothiazol-2-yl)-1-oxo-1H-pyrido[2,1-b][1,3]benzothiazole-4-carboxylate Refinement

**Table:**

Refinement on *F*^2^	Primary atom site location: dual
Least-squares matrix: full	Hydrogen site location: inferred from neighbouring sites
*R*[*F*^2^ > 2σ(*F*^2^)] = 0.039	H-atom parameters constrained
*wR*(*F*^2^) = 0.114	*w* = 1/[σ^2^(*F*_o_^2^) + (0.0599*P*)^2^ + 0.650*P*] where *P* = (*F*_o_^2^ + 2*F*_c_^2^)/3
*S* = 1.04	(Δ/σ)_max_ = 0.002
9507 reflections	Δρ_max_ = 1.62 e Å^−^^3^
254 parameters	Δρ_min_ = −0.48 e Å^−^^3^
0 restraints	

### Ethyl 2-(1,3-benzothiazol-2-yl)-1-oxo-1H-pyrido[2,1-b][1,3]benzothiazole-4-carboxylate Special details

**Table:**

Experimental. Data were collected at 110 K because the crystals cracked at 100K (presumably because of a phase change).

### Ethyl 2-(1,3-benzothiazol-2-yl)-1-oxo-1H-pyrido[2,1-b][1,3]benzothiazole-4-carboxylate Fractional atomic coordinates and isotropic or equivalent isotropic displacement parameters (Å^2^)

**Table:**

	*x*	*y*	*z*	*U*_iso_*/*U*_eq_	
C1	0.65788 (10)	0.69830 (7)	0.44909 (5)	0.01536 (12)	
O1	0.76314 (9)	0.65176 (6)	0.42328 (4)	0.02042 (12)	
C2	0.59709 (9)	0.66342 (7)	0.51720 (5)	0.01495 (12)	
C3	0.47917 (10)	0.72150 (7)	0.54192 (5)	0.01638 (13)	
H3	0.440918	0.695749	0.586835	0.020*	
C4	0.41390 (10)	0.81725 (7)	0.50297 (5)	0.01657 (13)	
C4A	0.47129 (10)	0.85341 (7)	0.43754 (5)	0.01602 (13)	
S5	0.41018 (3)	0.96920 (2)	0.38107 (2)	0.01898 (5)	
C5A	0.54333 (11)	0.94027 (7)	0.32026 (5)	0.01838 (14)	
C6	0.56482 (12)	1.00226 (8)	0.25492 (6)	0.02274 (16)	
H6	0.507318	1.068571	0.240780	0.027*	
C7	0.67208 (14)	0.96498 (9)	0.21098 (6)	0.02596 (18)	
H7	0.688512	1.005776	0.165996	0.031*	
C8	0.75624 (13)	0.86739 (9)	0.23270 (6)	0.02592 (18)	
H8	0.828854	0.842816	0.201681	0.031*	
C9	0.73690 (12)	0.80524 (8)	0.29827 (5)	0.02112 (15)	
H9	0.794908	0.739144	0.312423	0.025*	
C9A	0.62904 (10)	0.84346 (7)	0.34264 (5)	0.01668 (13)	
N9B	0.58738 (9)	0.79589 (6)	0.41105 (4)	0.01527 (11)	
C10	0.28474 (10)	0.87873 (8)	0.52649 (6)	0.01966 (14)	
O2	0.22737 (10)	0.96137 (7)	0.49221 (5)	0.02754 (15)	
O3	0.23929 (9)	0.83336 (6)	0.58949 (5)	0.02420 (14)	
C11	0.10238 (12)	0.88120 (9)	0.61358 (7)	0.02589 (18)	
H11A	0.091902	0.960214	0.597449	0.031*	
H11B	0.114066	0.877614	0.671879	0.031*	
C12	−0.03956 (13)	0.81867 (11)	0.57590 (8)	0.0330 (2)	
H12A	−0.055028	0.826992	0.518296	0.049*	
H12B	−0.130465	0.848089	0.594846	0.049*	
H12C	−0.026483	0.739813	0.589797	0.049*	
S1'	0.79989 (3)	0.47864 (2)	0.52878 (2)	0.01664 (5)	
C2'	0.65958 (10)	0.56342 (7)	0.55998 (5)	0.01534 (12)	
N3'	0.61155 (9)	0.53095 (6)	0.62404 (5)	0.01790 (12)	
C3A'	0.68446 (10)	0.43293 (7)	0.65194 (5)	0.01778 (13)	
C4'	0.66015 (12)	0.37627 (9)	0.72000 (6)	0.02291 (16)	
H4'	0.589405	0.404375	0.750868	0.027*	
C5'	0.74142 (12)	0.27854 (8)	0.74125 (6)	0.02500 (18)	
H5'	0.726025	0.239428	0.787114	0.030*	
C6'	0.84629 (12)	0.23661 (8)	0.69577 (6)	0.02418 (17)	
H6'	0.900282	0.169307	0.711295	0.029*	
C7'	0.87240 (12)	0.29179 (8)	0.62857 (6)	0.02156 (16)	
H7'	0.943451	0.263331	0.598000	0.026*	
C7A'	0.79064 (10)	0.39078 (7)	0.60724 (5)	0.01730 (13)	

### Ethyl 2-(1,3-benzothiazol-2-yl)-1-oxo-1H-pyrido[2,1-b][1,3]benzothiazole-4-carboxylate Atomic displacement parameters (Å^2^)

**Table:**

	*U*^11^	*U*^22^	*U*^33^	*U*^12^	*U*^13^	*U*^23^
C1	0.0185 (3)	0.0135 (3)	0.0143 (3)	0.0008 (2)	0.0034 (2)	0.0003 (2)
O1	0.0255 (3)	0.0185 (3)	0.0195 (3)	0.0057 (2)	0.0101 (2)	0.0022 (2)
C2	0.0164 (3)	0.0142 (3)	0.0145 (3)	0.0006 (2)	0.0037 (2)	0.0006 (2)
C3	0.0168 (3)	0.0163 (3)	0.0164 (3)	0.0004 (2)	0.0041 (2)	−0.0007 (2)
C4	0.0165 (3)	0.0153 (3)	0.0183 (3)	0.0010 (2)	0.0044 (2)	−0.0011 (2)
C4A	0.0167 (3)	0.0137 (3)	0.0175 (3)	0.0004 (2)	0.0027 (2)	−0.0007 (2)
S5	0.01982 (9)	0.01425 (8)	0.02237 (10)	0.00197 (6)	0.00266 (7)	0.00199 (6)
C5A	0.0204 (3)	0.0157 (3)	0.0182 (3)	−0.0009 (3)	0.0013 (3)	0.0020 (2)
C6	0.0265 (4)	0.0202 (4)	0.0202 (4)	−0.0012 (3)	0.0009 (3)	0.0060 (3)
C7	0.0330 (5)	0.0264 (4)	0.0182 (4)	−0.0024 (4)	0.0043 (3)	0.0068 (3)
C8	0.0328 (5)	0.0287 (4)	0.0178 (4)	0.0013 (4)	0.0088 (3)	0.0043 (3)
C9	0.0262 (4)	0.0218 (4)	0.0167 (3)	0.0022 (3)	0.0075 (3)	0.0027 (3)
C9A	0.0196 (3)	0.0158 (3)	0.0143 (3)	−0.0005 (2)	0.0024 (2)	0.0019 (2)
N9B	0.0177 (3)	0.0137 (3)	0.0146 (3)	0.0007 (2)	0.0036 (2)	0.0008 (2)
C10	0.0182 (3)	0.0187 (3)	0.0228 (4)	0.0012 (3)	0.0058 (3)	−0.0027 (3)
O2	0.0267 (3)	0.0243 (3)	0.0333 (4)	0.0094 (3)	0.0098 (3)	0.0033 (3)
O3	0.0225 (3)	0.0251 (3)	0.0283 (3)	0.0042 (2)	0.0130 (3)	0.0003 (3)
C11	0.0224 (4)	0.0283 (4)	0.0296 (5)	0.0026 (3)	0.0117 (3)	−0.0054 (4)
C12	0.0230 (4)	0.0314 (5)	0.0455 (6)	0.0005 (4)	0.0089 (4)	−0.0060 (5)
S1'	0.01858 (9)	0.01580 (8)	0.01603 (9)	0.00217 (6)	0.00447 (6)	0.00049 (6)
C2'	0.0165 (3)	0.0148 (3)	0.0150 (3)	0.0004 (2)	0.0034 (2)	0.0006 (2)
N3'	0.0191 (3)	0.0188 (3)	0.0166 (3)	0.0008 (2)	0.0054 (2)	0.0031 (2)
C3A'	0.0184 (3)	0.0178 (3)	0.0168 (3)	−0.0015 (3)	0.0023 (3)	0.0029 (2)
C4'	0.0245 (4)	0.0246 (4)	0.0196 (4)	−0.0026 (3)	0.0040 (3)	0.0064 (3)
C5'	0.0279 (4)	0.0224 (4)	0.0228 (4)	−0.0044 (3)	0.0000 (3)	0.0077 (3)
C6'	0.0276 (4)	0.0168 (3)	0.0247 (4)	−0.0015 (3)	−0.0038 (3)	0.0039 (3)
C7'	0.0240 (4)	0.0160 (3)	0.0223 (4)	0.0012 (3)	−0.0016 (3)	−0.0004 (3)
C7A'	0.0188 (3)	0.0153 (3)	0.0168 (3)	−0.0006 (2)	0.0008 (3)	0.0007 (2)

### Ethyl 2-(1,3-benzothiazol-2-yl)-1-oxo-1H-pyrido[2,1-b][1,3]benzothiazole-4-carboxylate Geometric parameters (Å, º)

**Table:**

C1—O1	1.2292 (10)	S1'—C2'	1.7553 (8)
C1—N9B	1.4267 (11)	C2'—N3'	1.3106 (11)
C1—C2	1.4374 (11)	N3'—C3A'	1.3831 (12)
C2—C3	1.3772 (11)	C3A'—C4'	1.4054 (12)
C2—C2'	1.4619 (11)	C3A'—C7A'	1.4067 (13)
C3—C4	1.3995 (12)	C4'—C5'	1.3869 (14)
C4—C4A	1.3859 (12)	C5'—C6'	1.4061 (16)
C4—C10	1.4688 (12)	C6'—C7'	1.3891 (14)
C4A—N9B	1.3750 (11)	C7'—C7A'	1.4021 (12)
C4A—S5	1.7250 (8)	C3—H3	0.9500
S5—C5A	1.7403 (10)	C6—H6	0.9500
C5A—C6	1.3912 (13)	C7—H7	0.9500
C5A—C9A	1.3994 (12)	C8—H8	0.9500
C6—C7	1.3851 (16)	C9—H9	0.9500
C7—C8	1.3988 (15)	C11—H11A	0.9900
C8—C9	1.3904 (13)	C11—H11B	0.9900
C9—C9A	1.3978 (13)	C12—H12A	0.9800
C9A—N9B	1.4168 (11)	C12—H12B	0.9800
C10—O2	1.2154 (12)	C12—H12C	0.9800
C10—O3	1.3392 (12)	C4'—H4'	0.9500
O3—C11	1.4566 (12)	C5'—H5'	0.9500
C11—C12	1.4909 (16)	C6'—H6'	0.9500
S1'—C7A'	1.7287 (9)	C7'—H7'	0.9500
			
O1—C1—N9B	119.82 (7)	N3'—C3A'—C7A'	115.21 (7)
O1—C1—C2	125.31 (7)	C4'—C3A'—C7A'	119.96 (8)
N9B—C1—C2	114.86 (7)	C5'—C4'—C3A'	118.74 (9)
C3—C2—C1	121.10 (7)	C4'—C5'—C6'	120.85 (9)
C3—C2—C2'	119.51 (7)	C7'—C6'—C5'	121.21 (9)
C1—C2—C2'	119.38 (7)	C6'—C7'—C7A'	117.94 (9)
C2—C3—C4	122.10 (8)	C7'—C7A'—C3A'	121.30 (8)
C4A—C4—C3	118.05 (7)	C7'—C7A'—S1'	128.87 (7)
C4A—C4—C10	118.69 (8)	C3A'—C7A'—S1'	109.83 (6)
C3—C4—C10	123.22 (8)	C2—C3—H3	119.0
N9B—C4A—C4	120.91 (7)	C4—C3—H3	119.0
N9B—C4A—S5	112.92 (6)	C7—C6—H6	120.8
C4—C4A—S5	126.17 (6)	C5A—C6—H6	120.8
C4A—S5—C5A	90.34 (4)	C6—C7—H7	119.9
C6—C5A—C9A	121.48 (9)	C8—C7—H7	119.9
C6—C5A—S5	125.86 (7)	C9—C8—H8	119.0
C9A—C5A—S5	112.64 (6)	C7—C8—H8	119.0
C7—C6—C5A	118.40 (9)	C8—C9—H9	121.2
C6—C7—C8	120.13 (9)	C9A—C9—H9	121.2
C9—C8—C7	122.03 (10)	O3—C11—H11A	109.7
C8—C9—C9A	117.63 (9)	C12—C11—H11A	109.7
C9—C9A—C5A	120.32 (8)	O3—C11—H11B	109.7
C9—C9A—N9B	128.75 (8)	C12—C11—H11B	109.7
C5A—C9A—N9B	110.92 (7)	H11A—C11—H11B	108.2
C4A—N9B—C9A	113.15 (7)	C11—C12—H12A	109.5
C4A—N9B—C1	122.97 (7)	C11—C12—H12B	109.5
C9A—N9B—C1	123.88 (7)	H12A—C12—H12B	109.5
O2—C10—O3	124.64 (9)	C11—C12—H12C	109.5
O2—C10—C4	123.14 (9)	H12A—C12—H12C	109.5
O3—C10—C4	112.22 (8)	H12B—C12—H12C	109.5
C10—O3—C11	117.00 (8)	C5'—C4'—H4'	120.6
O3—C11—C12	109.74 (9)	C3A'—C4'—H4'	120.6
C7A'—S1'—C2'	88.77 (4)	C4'—C5'—H5'	119.6
N3'—C2'—C2	121.37 (7)	C6'—C5'—H5'	119.6
N3'—C2'—S1'	115.89 (6)	C7'—C6'—H6'	119.4
C2—C2'—S1'	122.73 (6)	C5'—C6'—H6'	119.4
C2'—N3'—C3A'	110.29 (7)	C6'—C7'—H7'	121.0
N3'—C3A'—C4'	124.83 (9)	C7A'—C7'—H7'	121.0
			
O1—C1—C2—C3	179.64 (9)	O1—C1—N9B—C4A	−178.70 (8)
N9B—C1—C2—C3	0.31 (12)	C2—C1—N9B—C4A	0.66 (11)
O1—C1—C2—C2'	−1.53 (13)	O1—C1—N9B—C9A	1.28 (13)
N9B—C1—C2—C2'	179.14 (7)	C2—C1—N9B—C9A	−179.35 (7)
C1—C2—C3—C4	−0.66 (13)	C4A—C4—C10—O2	−1.47 (14)
C2'—C2—C3—C4	−179.49 (8)	C3—C4—C10—O2	−179.40 (9)
C2—C3—C4—C4A	0.03 (13)	C4A—C4—C10—O3	178.76 (8)
C2—C3—C4—C10	177.97 (8)	C3—C4—C10—O3	0.83 (13)
C3—C4—C4A—N9B	0.94 (12)	O2—C10—O3—C11	5.83 (15)
C10—C4—C4A—N9B	−177.10 (8)	C4—C10—O3—C11	−174.39 (8)
C3—C4—C4A—S5	−179.45 (7)	C10—O3—C11—C12	93.40 (12)
C10—C4—C4A—S5	2.51 (12)	C3—C2—C2'—N3'	−3.68 (13)
N9B—C4A—S5—C5A	−0.03 (7)	C1—C2—C2'—N3'	177.47 (8)
C4—C4A—S5—C5A	−179.66 (8)	C3—C2—C2'—S1'	175.35 (6)
C4A—S5—C5A—C6	179.73 (9)	C1—C2—C2'—S1'	−3.50 (11)
C4A—S5—C5A—C9A	1.02 (7)	C7A'—S1'—C2'—N3'	1.15 (7)
C9A—C5A—C6—C7	1.11 (14)	C7A'—S1'—C2'—C2	−177.93 (7)
S5—C5A—C6—C7	−177.50 (8)	C2—C2'—N3'—C3A'	178.20 (8)
C5A—C6—C7—C8	−0.21 (16)	S1'—C2'—N3'—C3A'	−0.89 (10)
C6—C7—C8—C9	−0.38 (17)	C2'—N3'—C3A'—C4'	179.46 (9)
C7—C8—C9—C9A	0.09 (16)	C2'—N3'—C3A'—C7A'	0.04 (11)
C8—C9—C9A—C5A	0.79 (14)	N3'—C3A'—C4'—C5'	−179.96 (9)
C8—C9—C9A—N9B	179.69 (9)	C7A'—C3A'—C4'—C5'	−0.57 (14)
C6—C5A—C9A—C9	−1.42 (14)	C3A'—C4'—C5'—C6'	0.02 (15)
S5—C5A—C9A—C9	177.36 (7)	C4'—C5'—C6'—C7'	0.33 (15)
C6—C5A—C9A—N9B	179.50 (8)	C5'—C6'—C7'—C7A'	−0.10 (14)
S5—C5A—C9A—N9B	−1.72 (9)	C6'—C7'—C7A'—C3A'	−0.46 (13)
C4—C4A—N9B—C9A	178.69 (8)	C6'—C7'—C7A'—S1'	178.88 (7)
S5—C4A—N9B—C9A	−0.96 (9)	N3'—C3A'—C7A'—C7'	−179.75 (8)
C4—C4A—N9B—C1	−1.32 (12)	C4'—C3A'—C7A'—C7'	0.80 (13)
S5—C4A—N9B—C1	179.02 (6)	N3'—C3A'—C7A'—S1'	0.80 (10)
C9—C9A—N9B—C4A	−177.26 (9)	C4'—C3A'—C7A'—S1'	−178.65 (7)
C5A—C9A—N9B—C4A	1.71 (10)	C2'—S1'—C7A'—C7'	179.58 (9)
C9—C9A—N9B—C1	2.75 (14)	C2'—S1'—C7A'—C3A'	−1.02 (7)
C5A—C9A—N9B—C1	−178.28 (7)		

### Ethyl 2-(1,3-benzothiazol-2-yl)-1-oxo-1H-pyrido[2,1-b][1,3]benzothiazole-4-carboxylate Hydrogen-bond geometry (Å, º)

**Table:**

*D*—H···*A*	*D*—H	H···*A*	*D*···*A*	*D*—H···*A*
C9—H9···O1	0.95	2.24	2.8116 (11)	118
C7—H7···O1^i^	0.95	2.42	3.3470 (12)	164

Symmetry code: (i) −*x*+3/2, *y*+1/2, −*z*+1/2.
